# Diffusion-weighted imaging versus short tau inversion recovery sequence: Usefulness in detection of active sacroiliitis and early diagnosis of axial spondyloarthritis

**DOI:** 10.1371/journal.pone.0201040

**Published:** 2018-08-07

**Authors:** Chiu Wai Shirley Chan, Helen Hoi Lun Tsang, Philip Hei Li, Kam Ho Lee, Chak Sing Lau, Pui Yan Stella Wong, Ho Yin Chung

**Affiliations:** 1 Division of Rheumatology and Clinical Immunology, The University of Hong Kong, Hong Kong, China; 2 Department of Radiology, Queen Mary Hospital, Hong Kong, China; 3 Division of Rheumatology, Tseung Kwan O Hospital, Hong Kong, China; University of Alberta, CANADA

## Abstract

**Objective:**

To compare the utility of Diffusion weighted imaging (DWI) with short tau inversion recovery (STIR) sequence in the diagnosis of early axial spondyloarthritis (SpA).

**Methods:**

Three hundred and five patients with chronic back pain were recruited consecutively from 3 rheumatology centers. Clinical, radiological and blood parameters were recorded. Patients with back pain duration no more than 3 years were classified as having early disease. STIR sequence and DWI of the sacroiliac joints were obtained and assessed using the Spondyloarthritis Research Consortium of Canada (SPARCC) method. The Assessment in Spondyloarthritis international Society definition was used to define positive STIR and DWI. Results were compared to expert diagnosed axial SpA.

**Results:**

When compared to STIR sequence, DWI had similar sensitivity (STIR 0.29, DWI 0.30) and specificity (STIR 0.97, DWI 0.92) in diagnosing sacroiliitis. However, STIR sequence had better reliability (STIR 0.78, DWI 0.61). In early disease group, DWI was not better than STIR sequence in detecting active sacroiliitis (sensitivity DWI vs STIR: 0.34 vs 0.36; specificity DWI vs STIR: 0.93 vs 0.93; positive predictive value DWI vs STIR: 0.92 vs 0.92; negative predictive value DWI vs STIR: 0.36 vs 0.37). Using the Assessment in SpondyloArthritis international Society (ASAS) classification criteria, 67/98 patients with early disease (sensitivity 0.91 specificity 0.90) and 221/305 overall (sensitivity 0.90; specificity 0.92) were classified as axial SpA. Among the expert diagnosed axial SpA patients who did not meet the ASAS criteria, only 2 had positive DWI.

**Conclusion:**

DWI and STIR have similar sensitivity in diagnosing axSpA in early disease. However, the use of DWI is limited by poorer reliability when compared with STIR.

## Introduction

Axial spondyloarthritis (SpA) describes a spectrum of chronic rheumatic diseases characterized by axial joint inflammation and ankylosis, of which ankylosing spondylitis (AS) is the prototype. It has significant social and psychiatric impacts [1, 2] and affects quality-of-life [3–5]. It is also associated with increased cardiovascular events [6, 7]. The introduction of various biologic therapeutic agents has led to dramatic improvements in the management of axial SpA by reducing symptoms and preserving functional status [8]. Early disease diagnosis is becoming more important as it will facilitate early therapeutic interventions.

Radiographic sacroiliitis is a key criterion in the diagnosis of AS. However, sole reliance on radiographs is associated with significant diagnostic delay [9]. Magnetic Resonance Imaging (MRI) can detect axial inflammation before radiographic changes, and has been widely used to diagnose axial SpA at an earlier stage.

More recently, diffusion-weighted imaging (DWI) has been proposed for the detection of sacroiliitis in axial SpA patients with early disease [10–11]. DWI, an imaging technique that quantifies the diffusivity of water molecules in a particular structure [12], has been suggested to be useful in detecting early disease inflammation. Despite this, DWI is reported to be no better than short tau inversion recovery (STIR) sequence in diagnosing axial SpA because of its low visual spatial resolution [13]. However, the utility of DWI in early disease stage has not been evaluated. By comparing with traditional STIR sequence, we aimed to evaluate whether DWI had better sensitivity in detecting sacroiliac (SI) joint inflammation in early axial SpA.

## Materials and methods

This is an on-going multicenter cohort evaluating the usefulness of DWI in the diagnosis and monitoring of axial SpA. Detailed methods have been described in our previous publication [13]. Data of patients recruited between March 2014 and July 2017 was analysed and presented in this report.

### Ethics approval

The study was approved by the institutional review board of The University of Hong Kong/ Hospital Authority Hong Kong West Cluster (institutional review board reference no. UW 14–085). It was conducted in accordance with the Declaration of Helsinki and the guidance of Good Clinical Practice, November 30, 2006.

### Patient recruitment

Three hundred and five patients with chronic back pain were consecutively recruited from 3 rheumatology centers in Hong Kong (Queen Mary Hospital, Pamela Youde Nethersole Eastern Hospital, and Tseung Kwan O Hospital), including patients who were referred for suspected inflammatory arthritis. Written informed consent was obtained from all studied patients. Inclusion criteria were: 1) low back pain for at least 3 months, ii) age greater than 18 years, iii) current back pain, and iv) ability to give written consent. Exclusion criteria were: i) pregnancy, ii) inability to undergo MRI examination, and iii) inability to give written consent.

### Clinical and demographic data

Clinical and demographic data were collected from the all recruited patients. They included age, sex, smoking, and drinking history, duration and characteristic of back pain, extra-spinal features and family history of SpA. Blood parameters included C-reactive protein (CRP), erythrocyte sedimentation rate (ESR) and human leucocyte antigen (HLA) B27. Lumbosacral (LS) spine radiographs were taken. Other assessments included the Bath Ankylosing Spondylitis Disease Activity Index (BASDAI) [14], Bath Ankylosing Spondylitis Functional Index (BASFI) [15], Bath Ankylosing Spondylitis Metrology Index (BASMI) [16], Bath Ankylosing Spondylitis Global score (BAS-G) [17], and the Ankylosing Spondylitis Disease Activity Score (ASDAS) [18] based on CRP or ESR.

### Radiographs of LS spine and SI joints grading

Radiographs of SI joints were graded according to the Modified New York (mNY) Criteria [19]: 0, normal; 1, doubtful; 2, obvious; 3, partial fusion; 4, complete fusion. Bilateral sacroiliitis grade 2 or above, or unilateral grade 3 or above was defined as radiological AS. All LS spine x-rays were graded by 2 readers (PHL, KHL) with disagreement resolved by consensus.

### Reading of MRI SI joints

All recruited patients underwent MRI examinations of the whole spine and SI joints. T1 weighted, T2 turbo spin echo (TSE), STIR images, and DWI were obtained simultaneously. Technical details of MRI have been reported previously [13] and are summarized in S1 Table. Free-breathing DWI with fat suppression was performed using a single-shot spin-echo echo-planar imaging sequence with 4 b-values (0, 100, 600, 1000s/ mm^2^). In this study, only the STIR image and DWI of the SI joints were used for analysis.

Blinded to the clinical data and other imaging sequences, STIR image and DWI of the SI joints were randomized and independently scored by 2 readers (CWSC, HYC) according to the spondyloarthritis research consortium of Canada MRI inflammation (SPARCC) scoring method [20]. Positive STIR images and DWI were defined as SPARCC score ≥ 2 by both readers. Negative STIR images and DWI were defined as SPARCC score <2 by either one or both readers.

### Expert opinion for axial SpA

Blinded to MRI and radiology results, an independent rheumatologist (HHLT) retrieved the patient diagnoses of axial SpA and non-axial SpA from available clinical records. Expert opinion was used as the “gold standard” for comparison of STIR images and DWI. It was also used as the “gold standard” to determine the performance of the Assessment of SpondyloArthritis International Society (ASAS) classification criteria for axial SpA with and without incorporating DWI of SI joints.

### Statistical analysis

Statistical analysis was performed using the commercial software of Statistical Package for the Social Sciences (SPSS) version 21. Baseline demographic and clinical characteristics were presented using descriptive statistics in mean and SD or as percentages. Agreement (Cohen’s kappa, ƙ) between readers regarding a positive DWI and STIR image was calculated. Agreement was defined as slight, fair, moderate, substantial, and almost perfect by the values of weighted Cohen’s kappa K<0.2, K = 0.2-<0.4, K = 0.4-<0.6, K = 0.6-<0.8, K = 0.8–1, respectively. Intra-class correlation coefficients were calculated between the SPARCC scores of 2 readers. Pearson’s correlation was used to test the correlation coefficient (cc) between the 2 readers. Positivity of the 2 images was compared to expert-diagnosed axial SpA for sensitivity, specificity, positive predictive value (PPV), negative predictive value (NPV), positive likelihood ratios (LR+) and negative likelihood ratios (LR-). A subgroup analysis was performed in the group with back pain less than 3 years.

## Results

All 305 patients with chronic back pain had MRI examinations performed. Two hundred and thirty-nine (78.4%) patients had a clinical diagnosis of axial SpA. Other diagnoses included degenerative spine disease (6.6%), non-specific back pain (NSBP) (5.8%), systemic lupus erythematosus with NSBP (1.6%), rheumatoid arthritis (3.6%), peripheral SpA (2.3%), osteoarthritis (<1%), fibromyalgia (<1%), gout (<1%) and synovitis-acne-pustulosis-hyperostosis-osteitis (SAPHO) syndrome (<1%). Patient’s characteristics are described in S2 Table.

Ninety-eight patients with back pain duration of no more than 3 years were classified as having early disease and the rest (n = 207) as having late disease. Our axial SpA patients had moderate to high disease activity, and moderate impairment of spinal mobility (S3 Table). The early disease group had a numerically better (although not statistically significant) functional performance than that in the late disease group. Among patients in the early disease group, 51/98 (52.0%) patients were HLA-B27 positive, 67/98 (68.4%) had rheumatologist-diagnosed axial SpA, and 36/98 (36.7%) had radiological AS based on mNY criteria. The mean disease duration in the early disease group was 1.3 years. In the late disease group, 129/207 (62.3%) patients were HLA-B27 positive, 169/207 (81.6%) had rheumatologist-diagnosed axial SpA, and 113/207 (54.6%) had radiological AS. The mean disease duration in the late disease group was 15.5 years.

### STIR image and DWI in sacroiliac joints

The inter-reader agreement of positive STIR and DWI was substantial (Cohen’s kappa values of STIR 0.78, DWI 0.61). The inter-reader reliability of SPARCC scores was excellent in both STIR image and DWI (intra-class correlation coefficient of STIR 0.96 and DWI 0.89). S3 and S4 Tables show the performance of STIR image and DWI respectively in detecting sacroiliitis in the early disease group, late disease group, and overall, using sensitivity, specificity, PPV, NPV, LR+, and LR-. The overall sensitivities of DWI were compatible to STIR sequence (DWI 0.30 vs STIR 0.29) but DWI had lower specificity (DWI 0.92 vs STIR 0.97) in diagnosing sacroiliitis. In the early disease group, STIR image and DWI had better sensitivity in diagnosing sacroiliitis. The specificities were similar.

### ASAS classification criteria for axial spondyloarthritis

S5 and S6 Tables show the performance of the ASAS classification criteria for axial SpA in our studied patients, including the imaging and clinical arms. The ASAS axial SpA criteria had very good sensitivity and specificity. When DWI was added as an adjunct in assessment, 7 of the patients who did not meet the ASAS criteria had positive DWI, of which only 2 had expert-diagnosed axial SpA. The use of DWI had a low yield in identifying active sacroiliitis in axial SpA patients who did not meet the ASAS classification criteria.

## Discussion

Early diagnosis of axial SpA is a challenge for rheumatologists. Clinical symptoms may be non-specific and radiographs may be normal for many years after disease onset. With the availability of effective therapeutic interventions, accurate and early diagnosis is vitally important to modify disease progression and decrease disease burden. MRI has been increasingly used to assess patients with suspected axial SpA. Although STIR and T1-weighted images are the 2 most widely used sequences, others such as DWI have been proposed to aid early diagnosis. To the best of our knowledge, this is the largest axial DWI cohort so far to evaluate its diagnostic ability in early axial SpA.

Similar to other studies [21, 22], we used expert opinion as “gold standard” for axial SpA diagnosis. Obviously, it would not be possible for us to use diagnostic criteria that include SI joints images for comparison. We also arbitrarily defined back pain duration of less than 3 years as early disease. This definition has also been used in another international cohort [23]. Despite the relatively short duration of symptoms, 36.7% of patients in the early disease group already had radiographic AS. Similar findings have been observed in other international cohorts [23, 24], which underscore the urgency in searching for a diagnostic tool useful in early disease. However, direct comparison among different cohorts is not possible due to differences in study design, patient recruitment and patient composition.

In the early disease group with disease duration less than 3 years, DWI has comparable diagnostic utility compared with STIR sequence in the detection of active sacroiliitis. Overall, DWI is limited by poorer reliability in diagnosing positive active sacroiliitis. Such findings can be accounted for by the poor visuospatial resolution of DWI (S1 Fig). As a result, the presence of structural damage in patients with long disease duration may influence the interpretation of the DWI SI joints images leading to misidentification of nonspecific signals as bone marrow edema (BME), hence compromising specificity. Overall, we showed that DWI has the ability to demonstrate active BME but is less useful than STIR sequence because of poorer reliability.

The possible role of DWI as a supplementary assessment in addition to the ASAS criteria was also evaluated in this study. In our cohort, among axial SpA patients who did not satisfy the ASAS criteria, only 2 patients had positive DWI. The ASAS criteria had very high sensitivity and specificity in our population, including the clinical arm, imaging and both arms. This may be due to higher pre-test likelihood in our cohort. In our local community, patients with chronic back pain already had additional clinical or radiological features before they were referred to the rheumatology clinics for specialist care. Therefore, in such settings, the addition of DWI in assessment has a low yield in identifying active sacroiliitis in axial SpA patients who did not meet the ASAS criteria.

We found that MRI active sacroiliitis is more common in early disease and in non-radiographic SpA. Compared with international MRI cohorts of patients with chronic back pain [25], our axial SpA patients have a similar rate of active sacroiliitis overall (29.3%) in STIR images, and a higher rate among patients with early disease (35.7%). Also STIR-detected active sacroiliitis occurs more often among non-radiographic axial SpA (33.7%) than in established ankylosing spondylitis (AS) (26.7%). We hypothesize that patients with established AS and long disease duration are prone to experience back pain attributable to other causes. This occurs in the absence of active sacroiliitis, rendering MRI more sensitive in early disease stage before radiographic changes.

It has been reported that BME, as defined in the ASAS criteria, can occur in patients with non-specific back pain as well as in healthy individuals [26]. In our study, we found similar findings that patients with degenerative spine can have positive STIR image (5%) as well as positive DWI (5%). However, we also found that positive DWI could be present in other rheumatic diseases, including 3 patients with rheumatoid arthritis (27.3%) and 1 with SLE (20%). Without consideration of other features of SpA, MRI alone is unsuitable for axial SpA diagnosis as it may lead to false positive results.

Previous studies proposed that apparent diffusion coefficient (ADC) values of the DWI SI joints might have a role in discriminating between axial SpA and degeneration [8, 9]. However, these studies measured the mean ADC (which included ADC value from both sacroiliitis lesion as well as normal-appearing bone marrow) which can be influenced by other factors such as age, bone marrow density, and the region of interest selected [27]. Therefore, care is required in the interpretation of these results. We have also previously evaluated the role of ADC with preliminary results showing that lesional ADC might have a role to differentiate disease activity and degeneration in the spine [12]. In our study, ADC evaluation of DWI of SI joints was not performed. Since the goal of our study was to evaluate diagnostic utility of DWI and ADC calculation would be based on BME in DWI of SI joints, detection of individual ADC would not have additional value to the diagnostic sensitivity. However, ADC might be useful in disease monitoring.

## Limitation and future prospects

This analysis is limited by its cross-sectional design. Our cohort mainly involved patients from specialty clinics who have a higher pre-test likelihood for axial SpA than those seen in primary care settings. We plan to follow up our patients with reassessment STIR and DWI for more comprehensive evaluation to delineate the possible role of DWI in disease monitoring.

## Conclusion

DWI and STIR have similar sensitivity and specificity in diagnosing axSpA. However, the use of DWI is limited by poorer reliability when compared with STIR.

## Supporting information

S1 Fig DWI have poorer visuospatial resolution. Left: STIR image of SI joints (TR/ TE 5000/80ms, field-of-view 150/240 mm^2^, matrix size 152x157); right: DWI SI joints (TR/TE 4000/90ms, field-of-view 300/241mm^2^, matrix size 124x100, b-value 100).
(DOCX)

S1 File Resubmission MRI data. (SAV)

S1 Table Imaging parameters for STIR and DWI sequences. (DOCX)

S2 Table Baseline characteristics of the study population. (DOCX)

S3 Table Positive and negative likelihood ratios, positive and negative predictive values, sensitivity and specificity of STIR- detected sacroiliitis in early disease group, late disease group and overall. (DOCX)

S4 Table Positive and negative likelihood ratios, positive and negative predictive values, sensitivity and specificity of DWI-detected sacroiliitis in early disease group, late disease group and overall. (DOCX)

S5 Table Sensitivity and specificity of the ASAS criteria and the addition of DWI in early disease group, late disease group and overall. (DOCX)

S6 Table Positive likelihood ratio and negative likelihood ratio of the ASAS criteria and the addition of DWI in early disease group, late disease group and overall. (DOCX)
