# Supplementary material for: Diffusion-weighted imaging versus short tau inversion recovery sequence: Usefulness in detection of active sacroiliitis and early diagnosis of axial spondyloarthritis
Source: PLoS One. 2018 Aug 7;13(8):e0201040. doi: 10.1371/journal.pone.0201040 (PMC6080754; doi:10.1371/journal.pone.0201040)
Supplement: S1 Fig — Left: STIR image of SI joints (TR/ TE 5000/80ms, field-of-view 150/240 mm2, matrix size 152x157); right: DWI SI joints (TR/TE 4000/90ms, field-of-view 300/241mm2, matrix size 124x100, b-value 100). (DOCX) [file pone.0201040.s001.docx]

| **Figure 1:** DWI have poorer visuospatial resolution. Left: STIR image of SI joints; right: DWI SI joints. |
| --- |
| 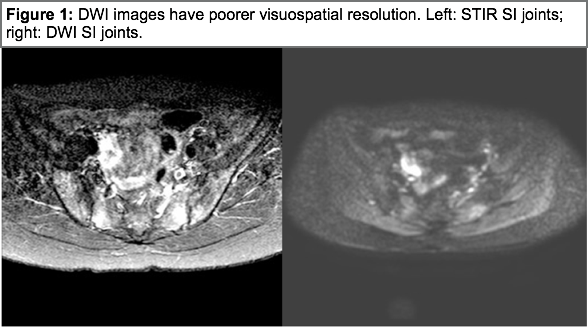 |
