# Supplementary material for: Diffusion-weighted imaging versus short tau inversion recovery sequence: Usefulness in detection of active sacroiliitis and early diagnosis of axial spondyloarthritis
Source: PLoS One. 2018 Aug 7;13(8):e0201040. doi: 10.1371/journal.pone.0201040 (PMC6080754; doi:10.1371/journal.pone.0201040)
Supplement: S1 Table — (DOCX) [file pone.0201040.s003.docx]

**S1 table.** Imaging parameters for STIR and DWI sequences

|  | STIR | DWI |
| --- | --- | --- |
| TR/TE (ms) | 5000/80 | 4000/90 |
| Field-of-view (mm^2^) | 150/240 | 300/241 |
| Matrix size | 152x157 | 124x100 |
| Slice thickness (mm) | 3.5 | 4 |
| SENSE factor | - | 2 |

TR, repetition time; TE, echo time; SENSE, sensitivity encoding; STIR, short tau inversion recovery; DWI, diffusion weighted imaging.
