# Supplementary material for: Diffusion-weighted imaging versus short tau inversion recovery sequence: Usefulness in detection of active sacroiliitis and early diagnosis of axial spondyloarthritis
Source: PLoS One. 2018 Aug 7;13(8):e0201040. doi: 10.1371/journal.pone.0201040 (PMC6080754; doi:10.1371/journal.pone.0201040)
Supplement: S2 Table — (DOCX) [file pone.0201040.s004.docx]

**S2 table:** Baseline characteristics of the study population

| Number | 305 |
| --- | --- |
| axSpA | 78.4% |
| Radiological AS | 49.8% |
| Age (years) | 44.1 ± 14.0 |
| Male | 48.9% |
| Smoker | 26.3% |
| Drinker | 10.2% |
| Duration of back pain (years) | 10.9 ± 11.0 |
| Back pain duration **≤** 3y | 32.1% |
| ASAS IBP | 58.8% |
| Family history of AS | 19.6% |
| HLA B27 | 65.2% |
| History of Uveitis | 25.8% |
| History of IBD | 2.3% |
| History of peripheral arthritis | 54.8% |
| History of enthesitis | 39.3% |
| History of dactylics | 9.7% |
| ESR (mm/hr) | 32.7 ± 25.5 |
| CRP (mg/dL) | 0.9 ± 1.8 |

axSpA, axial spondyloarthritis; AS, ankylosing spondylitis; ASAS, Assessment of SpondyloArthritis international Society; IBP, inflammatory back pain; HLA, human leucocyte antigen; IBD, inflammatory bowel disease; ESR erythrocyte sedimentation rate; CRP, C-reactive protein.
