# Supplementary material for: Diffusion-weighted imaging versus short tau inversion recovery sequence: Usefulness in detection of active sacroiliitis and early diagnosis of axial spondyloarthritis
Source: PLoS One. 2018 Aug 7;13(8):e0201040. doi: 10.1371/journal.pone.0201040 (PMC6080754; doi:10.1371/journal.pone.0201040)
Supplement: S3 Table — (DOCX) [file pone.0201040.s005.docx]

**S3 table:** Positive and negative likelihood ratios, positive and negative predictive values, sensitivity and specificity of STIR- detected sacroiliitis in early disease group, late disease group and overall.

|  | LR+  (95% CI) | LR-  (95% CI) | PPV  (95% CI) | NPV  (95% CI) | Sensitivity (95% CI) | Specificity (95% CI) | |
| --- | --- | --- | --- | --- | --- | --- | --- |
| Early disease | 5.00  (1.27, 19.72) | 0.69  (0.57, 0.85) | 0.93  (0.76, 0.98) | 0.37  (0.32, 0.41) | 0.36  (0.25, 0.48) | | 0.93  (0.77, 0.99) |
| Late disease | NA | 0.73  (0.67, 0.80) | 1.00 | 0.23  (0.21, 0.24) | 0.27  (0.20, 0.34) | | 1.00  (0.91, 1.00) |
| Overall | 9.52  (2.40, 37.80) | 0.73  (0.67, 0.80) | 0.97  (0.89, 0.99) | 0.27  (0.25, 0.29) | 0.29  (0.24, 0.36) | | 0.97  (0.89, 1.00) |

LR, likelihood ratio; PPV, positive predictive value; NPV, negative predictive value; CI, confidence interval.
