# Supplementary material for: Diffusion-weighted imaging versus short tau inversion recovery sequence: Usefulness in detection of active sacroiliitis and early diagnosis of axial spondyloarthritis
Source: PLoS One. 2018 Aug 7;13(8):e0201040. doi: 10.1371/journal.pone.0201040 (PMC6080754; doi:10.1371/journal.pone.0201040)
Supplement: S4 Table — (DOCX) [file pone.0201040.s006.docx]

**S4 table:** Positive and negative likelihood ratios, positive and negative predictive values, sensitivity and specificity of DWI-detected sacroiliitis in early disease group, late disease group and overall.

|  | LR+  (95% CI) | LR-  (95% CI) | PPV  (95% CI) | NPV  (95% CI) | Sensitivity (95% CI) | Specificity (95% CI) |
| --- | --- | --- | --- | --- | --- | --- |
| Early disease | 4.80  (1.21, 18.97) | 0.71  (0.58, 0.86) | 0.92  (0.75, 0.98) | 0.36  (0.32, 0.41) | 0.34  (0.23, 0.47) | 0.93  (0.77, 0.99) |
| Late disease | 3.54  (1.17, 10.80) | 0.78  (0.68, 0.89) | 0.94  (0.84, 0.98) | 0.22  (0.20, 0.25) | 0.29  (0.22, 0.36) | 0.92  (0.78, 0.98) |
| Overall | 3.95  (1.66, 9.37) | 0.75  (0.68, 0.84) | 0.94  (0.86, 0.97) | 0.27  (0.25, 0.29) | 0.30  (0.25, 0.37) | 0.92  (0.83, 0.97) |

LR, likelihood ratio; PPV, positive predictive value; NPV, negative predictive value; CI, confidence interval.
