# Supplementary material for: Diffusion-weighted imaging versus short tau inversion recovery sequence: Usefulness in detection of active sacroiliitis and early diagnosis of axial spondyloarthritis
Source: PLoS One. 2018 Aug 7;13(8):e0201040. doi: 10.1371/journal.pone.0201040 (PMC6080754; doi:10.1371/journal.pone.0201040)
Supplement: S5 Table — (DOCX) [file pone.0201040.s007.docx]

**S5 table:** Sensitivity and specificity of the ASAS criteria and the addition of DWI in early disease group, late disease group and overall.

|  | Sensitivity (95% CI) | Specificity (95% CI) |
| --- | --- | --- |
| **ASAS imaging** |  |  |
| - Early disease | 0.74 (0.62, 0.84) | 0.89 (0.72, 0.98) |
| - Late disease | 0.74 (0.67, 0.80) | 0.95 (0.81, 0.99) |
| - Overall | 0.74 (0.68, 0.79) | 0.92 (0.83, 0.98) |
| **ASAS clinical** |  |  |
| - Early disease | 0.53 (0.41, 0.65) | 1.00 (0.88, 1.00) |
| - Late disease | 0.61 (0.53, 0.68) | 1.00 (0.91, 1.00) |
| - Overall | 0.59 (0.52, 0.65) | 1.00 (0.95, 1.00) |
| **ASAS imaging +/- clinical** |  |  |
| - Early disease | 0.91 (0.82, 0.97) | 0.89 (0.72, 0.98) |
| - Late disease | 0.90 (0.84, 0.94) | 0.95 (0.82, 0.99) |
| - Overall | 0.90 (0.86, 0.94) | 0.92 (0.83, 0.97) |
| **ASAS imaging +/- clinical**  **+/- DWI** |  |  |
| - Early disease | 0.91 (0.82, 0.97) | 0.82 (0.63, 0.94) |
| - Late disease | 0.91 (0.86, 0.95) | 0.86 (0.71, 0.95) |
| - Overall | 0.91 (0.87, 0.95) | 0.85 (0.74, 0.92) |

CI, confidence interval; ASAS, Assessment of SpondyloArthritis international Society; DWI, diffusion weighted imaging.
