# Supplementary material for: Diffusion-weighted imaging versus short tau inversion recovery sequence: Usefulness in detection of active sacroiliitis and early diagnosis of axial spondyloarthritis
Source: PLoS One. 2018 Aug 7;13(8):e0201040. doi: 10.1371/journal.pone.0201040 (PMC6080754; doi:10.1371/journal.pone.0201040)
Supplement: S6 Table — (DOCX) [file pone.0201040.s008.docx]

**S6 table:** Positive likelihood ratio and negative likelihood ratio of the ASAS criteria and the addition of DWI in early disease group, late disease group and overall.

|  | LR+ (95% CI) | LR- (95% CI) |
| --- | --- | --- |
| **ASAS imaging** |  |  |
| - Early disease | 6.93 (2.36, 20.38) | 0.29 (0.19, 0.44) |
| - Late disease | 13.68 (3.54, 52.83) | 0.28 (0.21, 0.36) |
| - Overall | 9.63 (4.14, 22.42) | 0.28 (0.22, 0.35) |
| **ASAS clinical** |  |  |
| - Early disease | NA | 0.47 (0.37, 0.60) |
| - Late disease | NA | 0.39 (0.32, 0.47) |
| - Overall | NA | 0.41 (0.36, 0.48) |
| **ASAS imaging +/- clinical** |  |  |
| - Early disease | 8.53 (2.92, 24.92) | 0.10 (0.04, 0.21) |
| - Late disease | 16.64 (4.32, 64.11) | 0.11 (0.07, 0.17) |
| - Overall | 11.75 (5.06, 27.30) | 0.10 (0.07, 0.19) |
| **ASAS imaging +/- clinical**  **+/- DWI** |  |  |
| - Early disease | 5.12 (2.32, 11.37) | 0.10 (0.05, 0.23) |
| - Late disease | 6.74 (2.98, 15.26) | 0.10 (0.06, 0.17) |
| - Overall | 5.93 (3.35, 10.50) | 0.10 (0.07, 0.16) |

LR, likelihood ratio; CI, confidence interval; ASAS, Assessment of SpondyloArthritis international Society; DWI, diffusion weighted imaging.
